# Comparison of biological target volume metrics based on FDG PET-CT and 4DCT for primary non-small-cell lung cancer

**DOI:** 10.18632/oncotarget.18917

**Published:** 2017-07-01

**Authors:** Yingjie Zhang, Jianbin Li, Yili Duan, Wei Wang, Fengxiang Li, Qian Shao, Min Xu

**Affiliations:** ^1^ Department of Radiation Oncology, Shandong Cancer Hospital Affiliated to Shandong University, Jinan, China; ^2^ Shandong Academy of Medical Sciences, Jinan, China; ^3^ Changqing People’s Hospital, Jinan, China

**Keywords:** non-small cell lung cancer (NSCLC), four-dimensional computed tomography (4DCT), ^18^F-Fluorodeoxyglucose positron emission tomography CT (^18^F-FDG PET-CT), internal biological target volume (IBTV)

## Abstract

Fluorodeoxyglucose positron emission tomography-computed tomography (PET-CT) and four-dimensional CT (4DCT) are used in several methods for defining the biological target volume (BTV) in primary non-small cell lung cancer (NSCLC). Disagreements between the assessments using these methodologies make the use of BTV for radiotherapy planning controversial. In this study, we compared existing methods with our proposed internal biological target volume (IBTV) metric, derived by combining internal target volume (ITV) and BTV metrics. We defined the IBTV from ITV (IBTVi) or BTV (IBTVb) based on ITV or BTV with symmetrical margin expansion. We detected large differences between IBTV, IBTVi and IBTVb (*p* < 0.001), but no difference between ITV and BTV. A margin expansion of about 13 mm was necessary for ITV or BTV to encompass > 95% IBTV. The conformity index correlated negatively with IBTV/ITV, IBTV/BTV, IBTVi/ITV, and IBTVb/BTV volume ratios (*p* < 0.05). VR also increased the margins of IBTVi and IBTVb. Indeed, IBTV was much smaller than IBTVi or IBTVb, suggesting that using IBTV for radiotherapy planning could improve treatment by minimizing the radiation exposure of healthy tissue and organs surrounding tumors.

## INTRODUCTION

Positron emission tomography-computed tomography (PET-CT) is now widely used in the clinical practice of non-small cell lung cancer (NSCLC). PET-CT has revolutionized the field [1, 2], improving the definition of target volumes for radiotherapy planning [3]. Simulation of PET-CT scanning before radiotherapy is commonly advised because it provides more information than CT only. Taking advantage of the enhanced glucose metabolism of cancer cells, fluorodeoxyglucose (FDG) PET scanning distinguishes between normal and cancerous tissues, thereby accurately defines the biological target volume (BTV) [4]. Meanwhile, CT provides precise tumor localization, volume evaluation, and the extent of local tissue invasion [5]. However, the application of BTV in radiation therapy planning is controversial because of the poor spatial resolution of PET images, which adds uncertainty in tumor localization, and because of disagreements between the various methods used to define BTV.

To integrate PET images into the tumor target contouring process, many researchers have tried to compare BTV to gross tumor volumes (GTV) [6] and clinical tumor volumes (CTV) [7]. However, respiratory motion causes a mismatch between PET and CT analyses, contributing to the challenges in applying BTV [8, 9]. Using respiratory-averaged CT or maximum intensity projection (MIP) of 4DCT for attenuation correction showed smaller mismatch errors [9, 10]. Indeed, for thoracic cancers, BTV based on 4D PET-CT information is currently the best approach to delineate tumors [11].

Our previous study showed that BTV contoured by standardized uptake value (SUV) 2.0 or 20% of maximal SUV (SUVmax) approaches the internal target volume ITV_MIP_, while the spatial mismatch is obvious. Therefore, neither of them could replace ITV_MIP_ in spatial position and form [12]. The purpose of the current study was to determine which factors correlate with spatial mismatch between BTV and ITV, and to devise an applicable method to construct IBTV for radiotherapy planning.

## RESULTS

**Figure 1:**
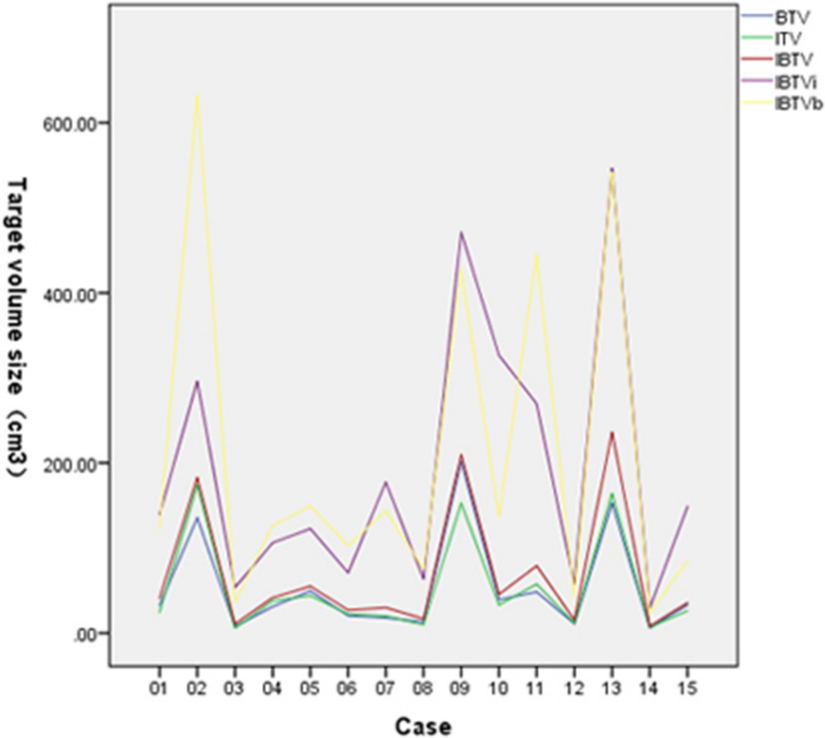
Personal target volume of BTV, ITV, IBTV, IBTVi and IBTVb

Figure 1 shows the variation of ITV, BTV, IBTV, IBTVi and IBTVb. The values of ITV, BTV, IBTV, IBTVi and IBTVb were 53.31 ± 59.89, 52.69 ± 59.21, 68.94 ± 75.68, 191.99 ± 158.12 and 206.24 ± 199.31 (cm^3^), respectively. There was no difference between ITV and BTV, whereas the difference between IBTV, IBTVi and IBTVb was significant (*p* < 0.001). Compared to IBTVi or IBTVb, IBTV was closer to ITV and BTV. A margin of 10.33 ± 4.03 mm (95% CI 8.10–12.56) was necessary for ITVi expanded from ITV, and a margin of 9.80 ± 4.36 mm (95% CI 7.38–12.22) was needed for ITVb expanded from BTV.

The VR values of IBTV to ITV and BTV, IBTVi to ITV, and IBTVb to BTV are listed in Table 1. We found that VR_IBTV/ITV_ and VR_IBTV/BTV_ were lower than VR_IBTVi/ITV_, VR_IBTVb/ITV_, VR_IBTVi/BTV_ and VR_IBTVb/BTV_ (*p* < 0.001). The CI of ITV and BTV was 52%±15%.

**Table 1:** Volume ratio of targets

	Mean	SD	95% lower limit	95% upper limit
**VR**_IBTV/ITV_^1^	1.38	0.22	1.26	1.50
**VR**_IBTVi/ITV_	5.10	2.53	3.70	6.50
**VR**_IBTV/BTV_	1.33	0.19	1.23	1.43
**VR**_IBTVb/BTV_	4.54	1.90	3.49	5.59

^1^VR: volume ratio.

The V value of tumor motion was 6.21 ± 2.97 mm. The D value between ITV and BTV, ITV and IBTV was 4.28 ± 3.32 mm, 2.11 ± 1.48 mm, respectively. Both of the latter values were lower than the corresponding V values (*p* = 0.001 and *p* < 0.001, respectively). The D value between ITV and BTV, ITV and IBTV showed no correlation with the V value (*p* > 0.05, respectively).

Table 2 illustrates the correlations of volume and displacement with IBTV, IBTVi and IBTVb. For each type of IBTV, VR was negatively correlated with CI. VR_IBTVi/ITV_ and VR_IBTVb/BTV_ also increased the margins of IBTVi and IBTVb. However, the factors related to displacement, such as V or D, were not correlated with CI or IBTVi and IBTVb margins. These results suggested that the difference between ITV and BTV was the main factor to decrease their concordance, and increase the margin expansion of IBTVi and IBTVb.

**Table 2:** A correlation analysis of the target volume and displacement

		V^1^	D_ITV-BTV_^2^	CI^3^	BTV^4^	ITV^5^	VR_IBTV/ITV_^6^	VR_IBTV/BTV_	VR_IBTVi/ITV_	VR_IBTVb/BTV_	M_ITV_^7^
D_ITV-BTV_	*r*	0.431									
	*p*	0.109	.								
CI	*r*	−0.139	−0.415								
	*p*	0.621	0.124	.							
BTV	*r*	0.193	−0.213	0.575							
	*p*	0.491	0.447	0.025	.						
ITV	*r*	0.307	−0.150	0.593	0.954						
	*p*	0.265	0.593	0.020	0.000	.					
VR_IBTV/ITV_	*r*	0.079	0.322	−0.732	−0.129	−0.318					
	*p*	0.781	0.242	0.002	0.648	0.248	.				
VR_IBTV/BTV_	*r*	0.346	0.565	−0.639	−0.336	−0.168	0.107				
	*p*	0.206	0.028	0.010	0.221	0.550	0.704	.			
VR_IBTVi/ITV_	*r*	−0.200	0.127	−0.704	−0.489	−0.621	0.718	0.125			
	*p*	0.475	0.652	0.003	0.064	0.013	0.003	0.657	.		
VR_IBTVb/BTV_	*r*	0.043	0.345	−0.657	−0.464	−0.336	0.179	0.682	0.193		
	*p*	0.879	0.208	0.008	0.081	0.221	0.524	0.005	0.491	.	
M_ITV_	*r*	0.184	−0.021	−0.298	0.271	0.101	0.647	−0.089	0.611	−0.220	
	*p*	0.511	0.941	0.280	0.329	0.720	0.009	0.754	0.016	0.430	.
M_BTV_^8^	*r*	0.399	0.227	−0.159	0.408	0.519	0.058	0.435	−0.247	0.565	0.031
	*p*	0.141	0.415	0.572	0.132	0.047	0.838	0.105	0.375	0.028	0.913

^1^V: the tumor motion vector.

^2^D_ITV-BTV_: the distance between ITV and BTV.

^3^CI: concordance index defined as the formula: CI (A/B) = (A∩B)/(AUB)

^4^BTV: biological target volume with threshold of SUV ≥ 2.0

^5^ITV: internal target volume composed of GTVs delineated on the 10 phases of four-dimensional CT.

^6^VR_A/B_: volume ratio of target A to B.

^7^M_ITV_: a margin from ITV expanding to ITVi.

^8^M_BTV_: a margin from BTV expanding to ITVb.

## DISCUSSION

Metabolic PET data is being widely used for radiotherapy target definition in NSCLC. Although many methods based on PET are currently used to define BTV, there is no consensus as to which method performs the best. Indeed, the various methods used yield widely varying estimations of the target volume [5, 10]. BTV is not well defined for radiotherapy planning, partially due to the inability to determine which BTV estimation method yields results that are closer to GTV, CTV, or ITV. Yu [6] and Meng [7] compared BTV to GTV and CTV, but this did not help to determine the spatial location. Even though PET and CT images can be obtained almost simultaneously, geometric mismatches between the targets contoured by PET and CT are frequent [13]. Recent studies have explored the usage of supplemental respiratory motion information and image correction based on 4DCT or 4D PET-CT [14–16]. Our previous study also showed obvious spatial mismatches between PET and CT targets [8]. To better combine metabolic and respiratory motion information, we constructed the IBTV metric by fusing ITV and BTV, or by expanding one of them.

Our study here showed that IBTVi or IBTVb was greater than IBTV. To contain 95% of the IBTV volume, an expansion of about 13 mm was necessary for ITV or BTV. This is similar to the results of Callahan Jason [17]. To contain 97% of ITV derived from 4D PET-CT MIP images, they suggested a 15 mm margin expansion from GTV to the planning target volume (PTV). Disadvantageously, the much greater margin would enlarge IBTVi and IBTVb. Notably, in our study IBTV was much smaller than IBTVi and IBTVb; therefore, using IBTV in radiotherapy planning might contribute to reduce the radiation dose for organs at risk.

Because ITV and BTV are similar in volume, VR_IBTV/ITV_ and VR_IBTV/BTV_ were also very close, with average values of 1.38 and 1.33, respectively. Molla *et al.* [18] reported a method similar to ours. They compared ITV values derived from 4D PET-CT and slow CT. They proposed ITVtotal as a new metric, obtained by combining slow CT and ITV4D, with a volume ratio of ITV4D/ITVtotal of 0.78. This ratio is slightly higher than our VR_BTV/IBTV_ ratio of 0.75. The difference might stem from their ITV4D consisting of BTVs of 8 phases.

**Figure 2:**
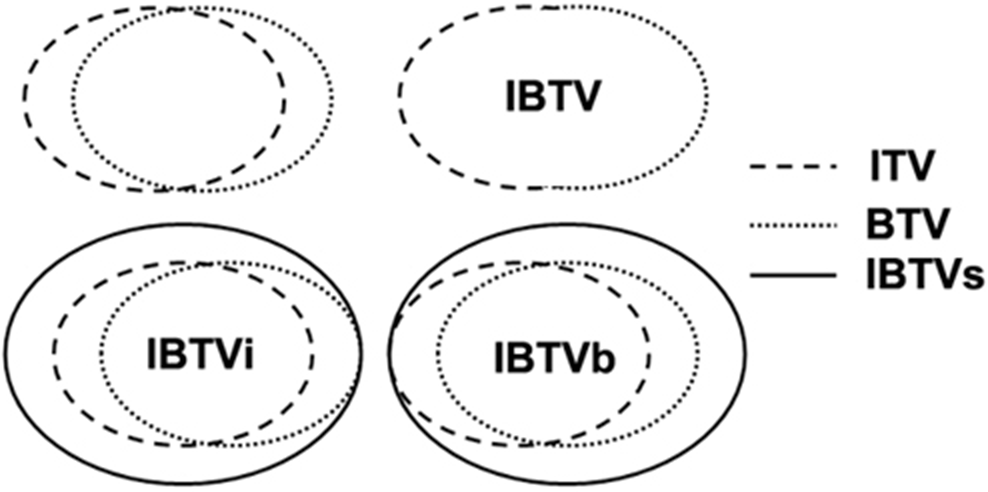
Schematic diagram to construct IBTVs by three metrics

We also analyzed mismatch factors between ITV and BTV. The V value was 6.21 ± 2.97 mm, which was greater than the D value between the centroid of BTV and ITV (4.28 ± 3.32 mm). D and V had no significant correlation, and they were not statistically related to CI, suggesting that neither V nor D was the main factor causing the mismatch between BTV and ITV. The CI of ITV and BTV was only 52% ± 15% in our study. Gondi reported a CI value of 0.44, which incorporated the target volumes of PET and 3DCT for NSCLC [19]. Thus, compared to 3DCT, 4DCT scanning can increase the matching index with PET. Our results showed a negative correlation between CI and VR_IBTV/ITV_, VR_IBTVi/ITV_, VR_IBTVi/ITV_, VR_IBTVb/BTV_. Therefore, CI was considered the main correlative factor increasing the size of IBTVs. We also found that CI was positively correlated with the size of BTV (*p* = 0.025) and ITV (*p* = 0.020), but did not correlate with V and D. One possible explanation is that tumors with larger size and higher T stage can more easily invade surrounding organs. As shown in Figure 2, the larger the tumor volume, the greater the difference observed between IBTV and IBTVi, IBTVb.

Traditionally, the PTVs expanded from IBTV, IBTVi, or IBTVb would be used in irradiation plans and the difference of geometric miss and radiation dose to adjacent risk organs would be analyzed to improve the treatment. However, in our study the size of IBTVi and IBTVb was approximately 3 times larger than that of IBTV, suggesting that verifying the difference in irradiation plans is unnecessary. Collectively, the IBTV obtained by combining BTV and ITV was smaller than IBTVi and IBTVb based on expansions from ITV and BTV, respectively. For larger tumors, we propose to compute PTV based on IBTV in order to reduce radiation dose to the surrounding organs at risk. Lastly, the impact of IBTV on radiotherapy planning and treatment outcomes needs to be further validated in the future studies.

## MATERIALS AND METHODS

### Patients

This study was approved by our ethics committee. From August 2013 to October 2014, 15 patients with proven primary NSCLC were eligible for 3D conformal radiotherapy or intensity-modulated radiotherapy in the Shandong Cancer Hospital Affiliated to Shandong University. All the patients were breathing freely and calmly during 4DCT and PET-CT simulation, and those with atelectasis or obstructive pneumonia were excluded. No patient underwent any treatment before simulation. Eleven patients were male and four were female, with a mean age of 67 years old (range from 45 to 84 years old). Five patients had centrally located lesions, and 10 patients had peripherally located lesions. Eight cases of adenocarcinoma, six cases of squamous cell carcinoma and one case of glandular squamous cell carcinoma were included. The primary tumor stages were T1 in three patients, T2 in eight patients, T3 in two patients and T4 in two patients. Detailed patient information is listed in Table 3.

**Table 3:** Patient characteristics and tumor volume variations

Case	Sex	Age (y)	Tumor location	History	Tumor Stage	SUV_max_^1^	GTV^2^ (cm^3^)	BTV^3^ (cm^3^)	ITV^4^ (cm^3^)
1	M	66	U^5^	A^7^	T2	25.51	16.67	32.75	24.50
2	F	68	L^6^	AS^8^	T3	11.00	142.89	135.47	173.72
3	M	70	U	A	T2	7.71	5.05	7.67	5.90
4	M	79	U	A	T1	8.80	26.94	31.11	37.66
5	F	49	L	S^9^	T2	8.85	38.70	49.45	44.01
6	M	66	U	S	T2	14.41	17.24	20.46	22.31
7	M	75	U	S	T2	12.39	14.53	18.07	19.99
8	F	65	U	A	T2	13.41	7.79	13.03	9.80
9	M	76	L	S	T4	15.13	109.84	201.56	152.80
10	M	84	L	A	T2	24.81	28.74	39.27	33.16
11	M	65	U	A	T4	14.38	40.06	48.46	57.53
12	F	67	U	A	T1	6.99	6.34	10.84	11.70
13	M	65	U	S	T4	14.52	154.12	152.57	164.10
14	M	45	U	A	T1	6.09	5.07	5.91	7.00
15	M	60	U	S	T3	11.34	20.35	33.02	26.15

^1^SUVmax: maximal standardized uptake value.

^2^GTV: gross target volume delineated on 3D-CT.

^3^BTV: biological target volume with threshold of SUV ≥ 2.0.

^4^ITV: internal target volume composed of GTVs delineated on the 10 phases of four-dimensional CT.

^5^U: Upper lobe.

^6^L: Lower lobe.

^7^A: Adenocarcinoma.

^8^AS: Adenosquamous carcinoma.

^9^S: Squamous carcinoma.

### Scanning protocol

All patients were immobilized using a thermoplastic mask in the supine position. For each patient, an axial enhanced 3DCT scan of the thoracic region was performed followed by an enhanced 4DCT scan using a 16-slice CT scanner (Philips Brilliance Bores CT, Koninklijke Philips N.V., Eindhoven, Netherlands). Subsequently, for each patient, an 18F-FDG PET-CT scan was performed using an integrated PET-CT scanner (Philips Gemini TF Big Bore) as described [12], with the patient placed in an identical simulation position as for the 3DCT and 4DCT scans.

### Image registration

All the 3DCT, 4DCT and PET-CT images were transferred to MIM imaging software (MIM-6.1.0, MIM Software Inc., Cleveland, OH, USA). An initial automatic rigid registration was performed with 3DCT and CT data of PET-CT. Because 3DCT and 4DCT images were obtained during the same imaging session, they were considered to register with each other automatically. Then, 4DCT and PET images were automatically registered to the same coordinate system. Finally, the registration was manually adjusted by matching bony anatomy such as the vertebral bodies, followed by judging and implementing of registration by the radiation oncologist. So all of the targets contoured on 4DCT and PET-CT images were reflected on the 3DCT images.

### Target volume definition

Target volumes were contoured based on 4DCT and PET-CT in the same coordinate system using MIM software. GTVs were manually contoured on 4DCT images with lung window setting (W = 1600, C = −600) and mediastinal window setting (W = 400, C = 40). One radiation oncologist contoured and another experienced senior radiation oncologist verified GTVs. By Boolean operation, ITV was composed of 10 phases of GTVs. Then, the BTV of the primary tumor was defined by calculating the ROI (region of interest) using a threshold of SUV ≥ 2.0 (SUV2.0) and the auto-contouring function of MIM. The BTV was verified manually to exclude adjacent normal tissues such as bone, heart and great vessels with the help of CT. IBTV was obtained by fusion of ITV and BTV. The IBTVi (or IBTVb) was defined based on ITV (or BTV) with a symmetrical margin expansion of 1 mm step by step in three dimensions until meeting the condition of [IBTVi(or IBTVb)∩IBTV]/IBTV ≥ 95%. The three metrics we used to construct IBTVs are represented in Figure 2.

### Target volumes comparison

We compared the position, size and concordance index (CI) of ITV, BTV and three types of IBTVs. The displacement of GTVs was measured by the shift of the centroid based on 10 phases of 4DCT. From hereon, we abbreviate the displacement range in left-right (LR), anterior-posterior (AP) and cranial-caudal (CC) directions as RLR, RAP and RCC, respectively. The 3D vectors of GTV centroids (V) were calculated according to the formula as V = (RLR^2^ + RAP^2^ + RCC^2^)^1/2^. We also abbreviate the distance between the centroid of ITV, BTV and IBTV in LR, AP and CC as DLR, DAP and DCC, respectively. The 3D distance (D) from ITV to BTV, ITV to IBTV, and BTV to IBTV was calculated as D = (DLR^2^ + DAP^2^ + DCC^2^)^1/2^. To compare the size of IBTV and ITV, IBTV and BTV, IBTVi and ITV, IBTVb and BTV we computed their volume ratios (VR); *i.e.*, the ratio of two target volumes. The spatial overlap of any two target volumes A and B is given by CI (A/B), defined as CI (A/B)=(A∩B)/(AUB).

### Statistical analysis

All data were analyzed using the SPSS software package (SPSS 23.0, SPSS Inc., Chicago, IL, USA). The one-way ANOVA test was used to compare tumor motion, volume and spatial overlap. Correlations were calculated using the Spearman test. All data with *P* < 0.05 were considered significant.

## Abbreviations

NSCLC: non-small cell lung cancer
PET-CT: positron emission tomography-computed tomography
4DCT: our-dimensional CT
BTV: biological target volume
ITV: internal target volume
IBTV: internal biological target volume
GTV: gross tumor volume
CTV: clinical tumor volume
PTV: planning target volume
MIP: maximum intensity projection
SUVmax: maximal SUV
CI: conformity index
VR: volume ratio
